# Ectoparasites associated with the Bushveld gerbil (*Gerbilliscus leucogaster*) and the role of the host and habitat in shaping ectoparasite diversity and infestations

**DOI:** 10.1017/S0031182023000562

**Published:** 2023-08

**Authors:** Amber T. Smith, Boris R. Krasnov, Ivan G. Horak, Eddie A. Ueckermann, Sonja Matthee

**Affiliations:** 1 Department of Conservation Ecology and Entomology, Stellenbosch University, 7602 Stellenbosch, South Africa; 2 Mitrani Department of Desert Ecology, Swiss Institute for Dryland Environmental and Energy Research, Jacob Blaustein Institutes for Desert Research, Ben-Gurion University of the Negev, Sede-Boqer Campus, 84990 Midreshet Ben-Gurion, Israel; 3 Department of Zoology and Entomology, Rhodes University, PO Box 94, Makhanda 6140, South Africa; 4 Unit for Environmental Sciences and Management, Potchefstroom Campus, North-West University, North-West, South Africa.

**Keywords:** Abundance, ectoparasites, *Gerbilliscus leucogaster*, habitat type, host factors

## Abstract

Rodents are known hosts for various ectoparasite taxa such as fleas, lice, ticks and mites. South Africa is recognized for its animal diversity, yet little is published about the parasite diversity associated with wild rodent species. By focusing on a wildlife-human/domestic animal interface, the study aims to record ectoparasite diversity and levels of infestations of the Bushveld gerbil, *Gerbilliscus leucogaster*, and to establish the relationship between ectoparasite infestation parameters and host- and habitat factors. Rodents (*n* = 127) were trapped in 2 habitat types (natural and agricultural) during 2014–2020. More than 6500 individuals of 32 epifaunistic species represented by 21 genera and belonging to 5 taxonomic groups (fleas, sucking lice, ticks, mesostigmatan mites and trombiculid mites) were collected. Mesostigmatan mites and lice were the most abundant and fleas and mesostigmatan mites the most prevalent groups. Flea and mesostigmatan mite numbers and mesostigmatan mite species richness was significantly higher on reproductively active male than female rodents. Only ticks were significantly associated with habitat type, with significantly higher tick numbers and more tick species on rodents in the natural compared to the agricultural habitat. We conclude that the level of infestation by ectoparasites closely associated with the host (fleas and mites) was affected by host-associated factors, while infestation by ectoparasite that spend most of their life in the external environment (ticks) was affected by habitat type.

## Graphical abstract

**Figure:**
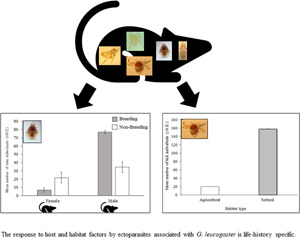

## Introduction

Small mammals including rodents play important roles in shaping ecological structure and species composition and diversity of plants within ecosystems (Nyirenda *et al*., 2020). They are often referred to as bio-engineers (Cameron, 2000; Reichman, 2007) because they contribute to the chemical and physical properties of soil (Galiano *et al*., 2014; Yong *et al*., 2019) and facilitate seed dispersal (Midgley and Anderson, 2005; Flores-Peredo *et al*., 2011) and pollination (Wiens *et al*., 1983; Wester *et al*., 2009). Small mammals also form an integral part of food webs by acting as a food source for predators as well as consumers of plant material and arthropods (Morand *et al*., 2006). In addition, rodents are known hosts for a diverse range of ectoparasite taxa (e.g. fleas, lice, ticks and mites) (Morand *et al*., 2006). Life history traits (e.g. group size, nesting behaviour and habitat use) of a rodent species often influences their exposure to parasites and therefore their parasite profiles (Vaumourin *et al*., 2015). This is because ectoparasites vary in their level of host specificity, micro-habitat preference and mode of transmission (Hopla *et al*., 1994; Paramasvaran *et al*., 2009). For example, lice are host-specific permanent parasites with all life stages occurring on the host's body and are transmitted through direct body contact between hosts, while ticks generally have a broader host range and attaches to a host only once during a life stage (larval, nymphal or adult) to obtain a bloodmeal (Morand *et al*., 2006). Several ectoparasite taxa are known vectors for disease-causing pathogens (e.g. *Yersinia pestis* for plague and *Rickettsia* species for various rickettsioses). Consequently, it is important to develop accurate parasite profiles for rodents that routinely move between natural (reserves) and anthropogenic (e.g. agriculture and village) habitats to prevent spillover of pathogens into human-associated habitats.

The occurrence of parasites and infestation within a host population are influenced by both host-associated and environment-associated factors (Krasnov and Matthee, 2010; Stanko *et al*., 2015; Obiegala *et al*., 2021). Host-associated factors include body size, age, sex and reproductive state (Morand and Poulin, 1998; Kołodziej-Sobocińska, 2019). For example, larger hosts can harbour more parasites due to larger total mass (more potential resources for parasites) and larger surface area (more space/niches for parasites) (Lindenfors *et al*., 2007; Froeschke *et al*., 2013). Body size is also indicative of host age and older hosts may accumulate parasites over time (Moore and Wilson, 2002; Poulin, 2007). Host sex can also influence parasite infestations, which are often related to sexual size dimorphism characteristic for many host species (Moore and Wilson, 2002) and difference in home range sizes between male and female hosts (Krasnov *et al*., 2005). In addition, elevated hormone levels can facilitate host sex-associated differences in mammals during the breeding season (Lightfoot, 2008). Reproductively active males that experience elevated testosterone levels, may become more aggressive towards conspecifics (Zielinski and Vandenbergh, 1993; Simon and Lu, 2006; Gleason *et al*., 2009) and enlarge their home range size in search of females (Tew and Macdonald, 1994; Bergallo and Magnusson, 2004). Testosterone has immunosuppressive properties that can increase male susceptibility to parasites (Hughes and Randolph, 2001; Klein, 2004; Matthee *et al*., 2010), whereas lowered immune defences during gestation can render female mammals more susceptible to parasites (Christe *et al*., 2000; Viljoen *et al*., 2011). Larger home ranges increase contacts between male hosts increasing probability of encounter with ticks and chiggers (Scantlebury *et al*., 2010; Butler *et al*., 2020). In contrast, during the breeding season, reproductively active female hosts are more tolerant of conspecifics and engage in social grooming between group members (Meaney and Stewart, 1979; Ganem and Bennett, 2004). A higher contact rate between host individuals can facilitate parasite exchange (Bordes *et al*., 2007; Patterson and Ruckstuhl, 2013), though host-induced mortality of parasites due to grooming can benefit female hosts (Marshall, 1981; Krasnov *et al*., 2002). In many rodent species, females may have a stronger nest association during the breeding season (Choate, 1972; Zenuto *et al*., 2001), which can promote infestations by nidicolous parasites such as fleas and mites (Krasnov *et al*., 2010). The effect of environment ( = habitat)-associated factors are first and foremost determined by high sensitivity of ectoparasites to air temperature and relative humidity in terms of, for example, development rate and survival (Krasnov *et al*., 2001; Herrmann and Gern, 2010; van der Mescht *et al*., 2013). This is particularly true for taxa with free-living life stages (fleas, mites and ticks). The vegetation structure (plant growth forms and vegetation cover) in a habitat can influence the microclimatic conditions by reducing the soil temperature and loss of soil moisture (He *et al*., 2010; Jucker *et al*., 2018; Lozano-Parra *et al*., 2018). Consequently, variation in the microclimatic conditions between habitat types (e.g. natural and transformed habitat types) can affect parasite occurrence and infestation levels (Lorch *et al*., 2007; Froeschke *et al*., 2013; Froeschke and Matthee, 2014; van der Mescht *et al*., 2016).

South Africa has a rich diversity of small mammals and, in particular, rodents (Skinner and Chimimba, 2005). Among the approximately 50 rodent species recorded in South Africa, many vary in geographic range and adaptability to habitat transformation (Skinner and Chimimba, 2005; Monadjem *et al*., 2015). Currently, most information on rodent parasites is limited to host-parasite lists in monographs of which some are outdated (Zumpt, 1961; Theiler, 1962; Ledger, 1980; Segerman, 1995; Horak *et al*., 2018). More recently, empirical studies based on large sample sizes have been conducted on a few rodent species (Matthee *et al*., 2007, 2010; Fagir *et al*., 2014, 2021; Stevens *et al*., 2022). These studies highlighted the potentially large ectoparasite diversity in locally abundant and regionally widespread species such as the four-striped mouse (*Rhabdomys pumilio*) (Matthee *et al*., 2007, 2010; Matthee and Krasnov, 2009), Namaqua rock mouse (*Micaelamys namaquensis*) (Fagir *et al*., 2014) and mole rats (Viljoen *et al*., 2011; Fagir *et al*., 2021) that readily adapt to agricultural habitats. In addition, the occurrence of undescribed ectoparasite species and new parasite-host and parasite-locality records in these studies suggested that the ectoparasite diversity in South African rodents is currently underestimated (Matthee *et al*., 2007; Matthee and Ueckermann, 2008, 2009; Fagir *et al*., 2014; Stevens *et al*., 2022). Moreover, ecological studies on factors that influence ectoparasite infestations and their species composition are sparse. In other words, ectoparasite communities of South African rodents and factors influencing structure of these communities remain to be further investigated.

The Bushveld gerbil (*Gerbilliscus leucogaster*) is a widespread, nocturnal rodent occurring mainly in the Grassland and Savanna biomes of southern Africa (Skinner and Chimimba, 2005; Odhiambo *et al*., 2008). These gerbils are also commonly found in agricultural areas where they are seen as pests of crops (Odhiambo *et al*., 2008; Von Maltitz *et al*., 2016). The species is medium in size (48–98 g) with no clear sexual dimorphism (Skinner and Chimimba, 2005; Lötter, 2010). It constructs burrows in sandy soils that are cleaned every night (Apps, 2012; Monadjem *et al*., 2015) and demonstrates communal living (De Graaff, 1981; Skinner and Chimimba, 2005), with family groups sharing burrows (Monadjem *et al*., 2015) and reproduce during spring-summer (Perrin and Swanepoel, 1987; Neal, 1991). Although the biology of this rodent is relatively well studied, limited data exist on the ectoparasite diversity associated with *G. leucogaster*. Current data for this species is, as mentioned above, restricted to historic monographs (Zumpt, 1961; Ledger, 1980; De Graaff, 1981; Segerman, 1995; Horak *et al*., 2018) and a single field study at a single locality in the Savanna biome (Braack *et al*., 1996). The latter study identified a number of louse, flea and tick species on *G. leucogaster*, but mite species identification was incomplete.

Here, we studied ectoparasite diversity and factors that drive their infestation on *G. leucogaster*. Our objectives were (a) to record ectoparasite (especially mite due to incomplete knowledge) species and their level of infestation (mean abundance and prevalence) and (b) to establish the relationship between host-related (sex, body size and reproductive state) and habitat-related (natural *vs* agricultural) factors on ectoparasite infracommunity structure, namely (a) diversity in terms of species richness and (b) abundance in terms of the number of ectoparasite individuals. Given the nest type, social behaviour and habitat use of *G. leucogaster*, we expected high ectoparasite, especially mite, diversity and abundance.

## Materials and methods

### Study area

**Figure 1.**
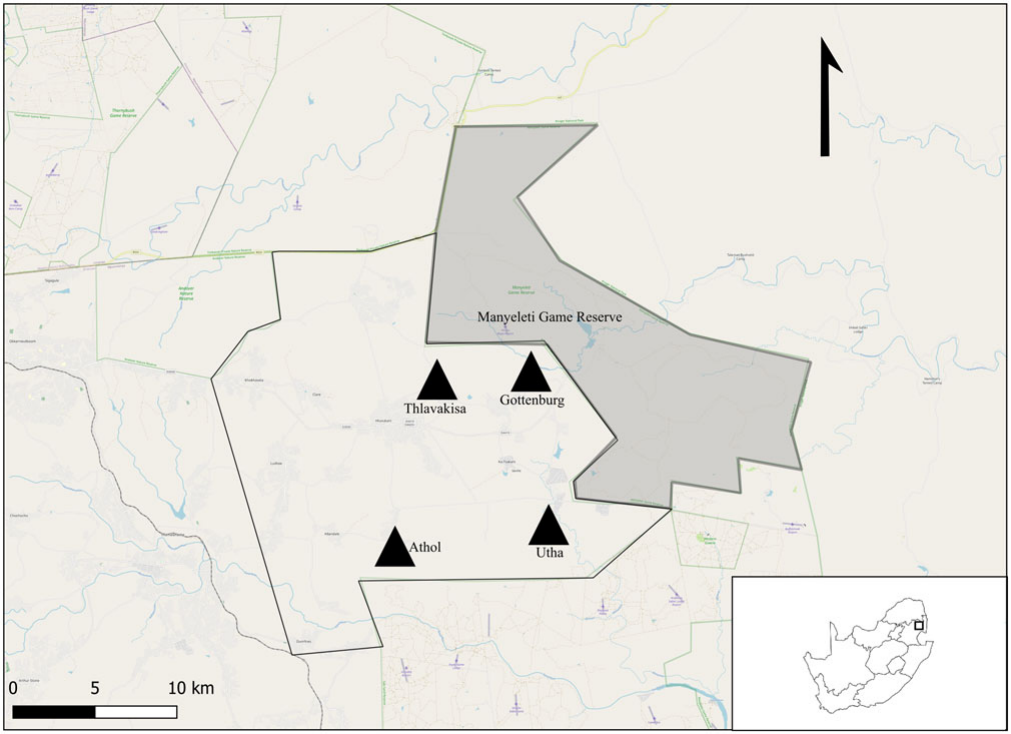
Locality map of the study area for *Gerbilliscus leucogaster* (*n* = 127) within the Mnisi OneHealth platform in the Mpumalanga Province, South Africa. The village sites are represented by black triangles (*n* = 4) and the shaded area is Manyeleti nature reserve.

The study is part of a larger on-going research programme conducted within the Mnisi OneHealth platform in Mpumalanga Province, South Africa (Berrian *et al*., 2016) (Fig. 1). The Mnisi community comprises several villages that are bordered by large, fenced nature reserves. Rodents were trapped in 4 villages and 4 crop fields within these villages' (Gottenburg 24°38′01″ S, 31°25′19″ E; Thlavekisa 24°37′51″ S, 31°22′42″ E, Athol 24°42′29″ S, 31°20′43″ E and Utah 24°50′14″ S, 31°02′45″ E). In general, subsistence farming is practiced within these settlements. Small vegetable patches and cattle occur on the property (village), while small crop fields (agricultural areas) often occur between the village and the nature reserve. Rodents were also trapped at 4 localities in the Manyeleti Nature Reserve (24°38′52″ S, 31°31′35″ E) that represents pristine natural Savanna vegetation.

### Rodent trapping

*Gerbilliscus leucogaster* individuals were trapped at 3 villages and their respective crops in (August–October) of 2014 and 2015 and once in summer (January) of 2015 and at 4 villages and their respective crops in spring (August–October) 2019 and 2020 (Fig. 1). All localities were >1 km apart. In each habitat type, a standardized rodent trap design was used. Sherman-type live traps (80) were set in trap lines baited with a mixture of peanut butter and oats and set for 3–4 days per locality. Each locality was only trapped once during each trap period. During this time, traps were checked twice a day and closed in the heat of the day (10:00–15:00). Targeted rodents were removed from traps, placed in pre-marked plastic bags and euthanized with Isoflurane. Once labelled, the carcasses were frozen at −20°C (to preserve the integrity of the material and to kill the ectoparasites) for later examination. The study was approved by the Animal Ethics Committees of Stellenbosch University (Reference numbers: ACU 2016-00190; ACU 2018-4555; ACU 2020-17062), the Mpumalanga Tourism and Parks Agency (Permit number ES 5/14; MPB. 5694; MPB. 5663), Department of Agriculture, Forestry and Fisheries (Reference number 12/11/1/7/5) and Pretoria University (Reference number V046-14; VO23-19).

### Laboratory procedures

Prior to ectoparasite removal, the carcasses were thawed. Each rodent body was systematically (anteriorly and posteriorly) examined for ectoparasites using a Zeiss Stemi DV4 stereomicroscope (Carl Zeiss Light Microscopy, Göttingen, Germany) and fine point forceps (used to separate hair shafts and to remove the ectoparasites). All rodents were preliminarily identified based on morphological and dental morphology using taxonomic references (De Graaff, 1981; Perrin and Swanepoel, 1987; Skinner and Chimimba, 2005; Monadjem *et al*., 2015) and thereafter confirmed molecularly (cytochrome b gene) (Bastos *et al*., 2011). For each rodent individual, we recorded sex and body measurements (weight, total body length, tail length and hind foot length). Thereafter reproductive state (reproductively active females were characterized by a perforated vagina and reproductively active males were characterized by enlarged testes) was recorded. All fleas, lice, ticks and mesostigmatan mites (hereafter referred to as mites) were removed and transferred to sample tubes filled with 100% ethanol. All fleas were counted (male and female) but only male individuals were available for identification and used for the species abundance (this is because female fleas were used in a separate project). For each louse species counts of immature stages were combined and reported as nymphs. In the case of chiggers, only a sub-sample was collected and the parasitope (region on the body where chiggers occurred) recorded. Fleas (males), lice, mites and chiggers were cleared, and slide mounted (fleas in Canada balsam and the rest in Hoyer's mounting medium) using standard techniques, while ticks remained unmounted. Ectoparasite identification was conducted using taxonomic reference keys. Fleas were identified according to Segerman (1995) and lice were identified using various reference sources (Ledger, 1980; Durden and Musser, 1994). Ticks were identified according to Walker *et al*. (2000) and Horak *et al*. (2018). Mites were identified using various reference sources (Till, 1963; Herrin and Tipton, 1975; Matthee and Ueckermann, 2008), but species identification was not possible for all mites. Chiggers were identified following Zumpt (1961) and Stekolnikov and Matthee (2019). Identification of lice, fleas and chiggers was done using a Leica DM1000 light microscope (Leica Microsystems GmbH, Wetzlar, Germany) and that of ticks using a Leica MZ75 high-performance stereomicroscope (Leica Microsystems GmbH, Wetzlar, Germany).

### Data analysis

Rodent and ectoparasite data were pooled per locality within each of the habitat types (natural, agriculture and village) within a sampling year. We divided the ectoparasites into higher taxonomic groups (fleas, lice, mites and ticks) and pooled the different life stages (i.e. larvae, nymphs, males and females) within the respective ectoparasite taxa. For each higher ectoparasite taxon, we calculated mean abundance (mean number of parasites on an individual host) and prevalence (% of hosts infested). In addition, we considered total counts of ectoparasites of a given higher taxon and their species richness (the number of species) on an individual host (i.e. infracommunity). Analysis of species richness was not carried out for fleas and lice because these taxa were dominated by 1 and 2 species, respectively, even though more than one species were recorded. Because (a) the data were collected in different years and (b) some dependent variables (the number of ectoparasite individuals and ectoparasite species richness) were not normally distributed (Shapiro–Wilk tests), we applied generalized linear mixed-effect models with the *lme4* package (Bates *et al*., 2015) in R (R Core Team, 2020) using year of sampling as a random factor and a negative-binomial error distribution. Thereafter, we applied model selection and model averaging using the R package *MuMIn* (Bartoń, 2018) to identify host-associated (sex, body size using tail length as a proxy and reproductive state) and habitat-associated (habitat type) variables and the interactions between them affecting the numbers of ectoparasite individuals and species. For each model averaging scenario, 95% confidence interval values are reported, and explanatory variables were considered significant when confidence intervals did not include zero. We also calculated conditional R^2^ (the proportion of the variance explained by both fixed and random effects) and marginal R^2^ (the proportion of the total variance explained by the fixed effects) of Nakagawa *et al*. (2017) for all models.

## Results

A total of 127 *G. leucogaster* (77 males and 50 females) were captured and examined for ectoparasites (Table 1). The average tail length was 13.98 ± 0.20 cm in males and 14.02 ± 0.26 cm in females. Data on reproductive state were available for 123 rodents of which 74 were reproductively active (61 males and 13 females). We identified 21 genera that comprised of 28 ectoparasitic and 4 non-parasitic species (predatory mites: mites that predate on invertebrates in the nest of the host) (Table 2). Mites were represented by 14 species, followed by 9 chigger species, 5 tick species, 3 flea species and 2 louse species (Table 2). In total, 6758 epifaunistic individuals (excluding chiggers) were recorded of which mites and lice were the most abundant. Fleas were the most prevalent followed by mites (Table 3).

**Table 1.** Sampling period and sample size for *Gerbilliscus leucogaster* (*n* = 127) trapped in Mpumalanga, South Africa (2014–2020)

Month and Year	No. of animals	Males	Females
August 2014	15	5	10
January 2015	14	7	7
September 2015	15	12	3
September 2019	23	16	7
October 2020	60	37	23
Total	**127**	**77**	**50**

**Table 2.** Epifaunistic arthropod taxa recorded on *Gerbilliscus leucogaster* (*n* = 127) in Mpumalanga, South Africa, 2014–2020

	Order	Suborder	Family/Subfamily	Taxon
Fleas	Siphonaptera		**Pulicidae**	
			Xenopsyllinae	*Xenopsylla bechuanae* de Meillon, 1947
				*X. brasiliensis* (Baker), 1904
				*X. frayi* de Meillon, 1947
Lice	Phthiraptera	Anoplura	**Hoplopleuridae**	*Hoplopleura biseriata* Ferris, 1921
			**Polyplacidae**	*Polyplax biseriata* Ferris, 1923
Ticks	Parasitiformes	Ixodida	**Ixodidae**	
			Haemaphysalinae	*Haemaphysalis elliptica* group
				*Hae. spinulosa* group
			Hyalomminae	*Hyalomma truncatum* Koch, 1844
			Rhipicephalinae	*Dermacentor rhinocerinus* (Denny, 1843)
				*Rhipicephalus follis/simus* group
Mites	Parasitiformes	Mesostigmata	**Laelapidae**	
			Laelapinae	*Androlaelaps marshalli* Berlese, 1911
				*A. oliffi* (Zumpt & Patterson, 1951)
				*A. taterae* (Zumpt & Patterson, 1951)
				*A. theseus* Zumpt, 1950
				*Laelaps liberiensis* Hirst, 1925
				*L. muricola* Trägårdh, 1910
				*L. simillimus* Zumpt, 1950
			**Macronyssidae**	*Ornithonyssus bacoti* (Hirst, 1930)
			**Pachylaelapidae**	*Pachylaelaps* sp.^P^
			**Uropodidae**	Uropodidae sp.^P^
	Sarcoptiformes	Acaridia	**Acaroidae**	Acaroidae sp.^P^
		Psoroptidia	**Atopomelidae**	*Listrophoroides (Afrolistrophoroides) mastomys* Radford, 1940
	Trombidiformes	Prostigmata	**Cheyletidae**	*Cheyletus zumpti* Fain, 1972^P^
			**Myobiidae**	*Austromyobia forcipifer* Lawrence, 1954
			**Trombiculidae**	
			Gahrliepiinae	*Gahrliepia nana* (Oudemans, 1910)
			Trombiculinae	*Ascoschoengastia ueckermanni* Stekolnikov and Matthee, 2019
				*Microtrombicula mastomyia* (Radford, 1942)
				*Microtrombicula* sp.
				*Schoutedenichia dutoiti* (Radford, 1948)
				*S. horaki* Stekolnikov and Matthee, 2019
				*S. lumsdeni* Vercammen-Grandjean, 1958
				*S. morosi* Vercammen-Grandjean, 1958
				*Trombicula walkerae* Stekolnikov and Matthee, 2019

Family name indicated in bold. Taxonomic authority included.

^P^Predatory feeding strategy.

**Table 3.** Epifaunistic arthropod taxa and their infestation parameters recorded from *Gerbilliscus leucogaster* (*n* = 127) in Mpumalanga Province, South Africa, 2014–2020

Ectoparasite taxa	Total Abundance	Adults (%)	Larvae (%)	Nymphs (%)	Adult Male (%)	Adult Female (%)	Sex Ratio (♂:♀)	Mean Abundance (±SE)	Prevalence (%)
Fleas^a^	**875**	**100**	–	–	**48.11**	**58.89**	**1:1.08**	**6.89 ± 0.69**	**87**.**40**
*Xenopsylla bechuanae*^b^	2	100	–	–	–	–	–	0.02 ± 0.01	1.57
*X. brasiliensis*^b^	7	100	–	–	–	–	–	0.06 ± 0.03	3.15
*X. frayi*^b^	412	100	–	–	–	–	–	3.24 ± 0.35	75.59
Lice	**1819**	**49.26**	–	**50.74**	**24.41**	**24.84**	**1:1.02**	**14.32 ± 1.93**	**76**.**38**
*Hoplopleura biseriata*	4	100	–	0	75	25	3:1	0.03 ± 0.01	2.67
*Polyplax biseriata*	1815	49.15	–	50.85	24.30	24.85	1:1.02	14.33 ± 1.93	74.80
Ticks	**177**	–	**43.50**	**56.50**	–	–	–	**1.39 ± 0.37**	**32**.**28**
*Dermacentor rhinocerinus*	3	–	33.33	66.67	–	–	–	0.02 ± 0.01	2.36
*Haemaphysalis elliptica* group	20	–	25	75	–	–	–	0.16 ± 0.05	9.45
*Hae. spinulosa* group	4	–	50	50	–	–	–	0.03 ± 0.02	1.57
*Hyalomma truncatum*	111	–	36.04	63.96	–	–	–	0.87 ± 0.30	22.05
*Rhipicephalus follis/simus* group	39	–	74.36	25.64	–	–	–	0.31 ± 0.20	3.94
Mites	**3887**	**73.73**	**0**	**26.27**^c^	**29.74**	**43.99**	**1:1.48**	**30.61 ± 3.83**	**79**.**53**
*Androlaelaps marshalli*	458	91.48	0	8.52	13.54	77.95	1:6.05	3.61 ± 0.50	57.48
*A. oliffi*	2821	68.81	0	31.19	37.26	31.55	1.18:1	22.21 ± 3.49	69.29
*A. taterae*	13	100	0	0	7.69	92.31	1:12	0.10 ± 0.05	5.51
*A. theseus*	199	93.47	0	6.53	5.03	88.44	1:17.60	1.57 ± 0.31	32.28
*Laelaps liberiensis*	17	82.35	0	17.65	17.65	64.71	1:3.67	0.13 ± 0.08	4.72
*L. muricola*	1	100	0	0	0	100	–	0.01 ± 0.01	0.79
*L. simillimus*	19	78.95	0	21.05	26.32	52.63	1:2	0.15 ± 0.12	1.57
*Ornithonyssus bacoti*	1	0	0	100	0	0	–	0.01 ± 0.01	0.79
*Pachylaelaps* sp.^P^	12	100	0	0	16.67	83.33	1:7	0.09 ± 0.03	7.09
Uropodidae sp.^P^	27	55.56	0	44.44	0	55.56	–	0.42 ± 0.10	8.66
Acaroidae sp.^P^	61	0	0	100	0	0	–	0.95 ± 0.47	1.57
*Listrophoroides (A.) mastomys*	104	92.31	0	7.69	21.15	71.15	1:3.36	0.82 ± 0.45	4.72
*Cheyletus zumpti* ^P^	149	100	0	0	0	100	–	1.17 ± 0.29	22.05
*Austromyobia forcipifer*	5	100	0	0	0	100	–	0.04 ± 0.02	3.15
Chiggers	–	–	–	–	–	–	–	–	**35**.**43**

Number/proportions for ectoparasite groups are indicated in bold.

a Taxon count includes all male and female individuals.

b Count for flea species based on male individuals only, lice nymphs: instars I, II and III combined.

c Mite nymphs represents proto- and deutronymphs.

P Predatory feeding strategy.

### Epifaunistic diversity

Three flea species from the genus *Xenopsylla* were recovered from *G. leucogaster* (Table 3). Based on adult male fleas, *Xenopsylla frayi* was the most abundant and prevalent (>70%) flea species. The 2 remaining species (*Xenopsylla brasiliensis and Xenopsylla bechuanae*) occurred on <5% of the rodents (Table 3). Among lice, *Polyplax biseriata* was the most prevalent (74.80%) compared to *Hoplopleura biseriata* (2.67%) (Table 3). Five ixodid tick species, from 4 genera, were recorded (Table 3). *Hyalomma truncatum* was the most abundant and prevalent, followed by *Dermacentor rhinocerinus*. Ticks were represented by nymph and larval life stages (Table 3). Fourteen mite species (excluding Trombiculidae) were found. *Androlaelaps oliffi* was the most abundant and prevalent species, followed by *Androlaelaps marshalli*. *Androlaelaps* mites represented 90% of the 14 mite species. Three unknown mite species were recorded (1 in the genus *Pachylaelaps* and 1 each from families Acaroidae and Uropodidae). *Cheyletus zumpti* was the most prevalent (22.05%) predatory mite. The adult female life stage was the most common life stage for 6 of the Laelapidae species (Table 3). Nine chigger species were recorded with an overall prevalence of 35.43% (Table 3). *Schoutedenichia lumsdeni* was the most prevalent species followed by *Gahrliepia nana*. Chiggers occurred on various parasitopes of which the pinna (external part of ear) was the most preferred followed by the front leg (Table 4).

**Table 4.** Prevalence and parasitope for chigger species (Trombiculidae) recorded from *Gerbilliscus leucogaster* (*n* = 127) in Mpumalanga Province, South Africa, 2014–2020.

Chigger species	Prevalence (%)	Parasitope
*Ascoschoengastia ueckermanni*	0.79	pinna
*Gahrliepia nana*	7.87	tail base, perineum
*Microtrombicula mastomyia*	1.57	pinna
*Microtrombicula* sp.	0.79	front leg
*Schoutedenichia dutoiti*	3.15	pinna, front leg
*S. horaki*	3.15	face, front leg, hind leg
*S. lumsdeni*	10.24	pinna
*S. morosi*	6.30	pinna, front leg, face, perineum
*Trombicula walkerae*	1.57	pinna

### Effects of host- and habitat-associated factors

**Figure 2.**
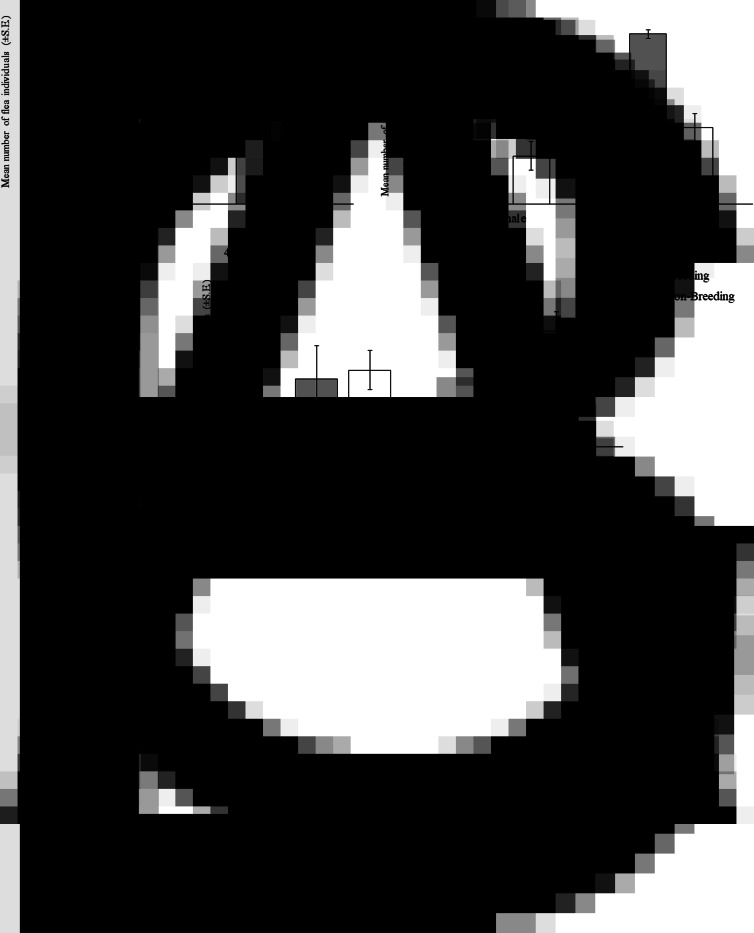
Mean number of: (A) flea individuals (±s.e.), (B) mite individuals (±s.e.) and (C) mite species (±s.e.) per host sex and per reproductive state for *Gerbilliscus leucogaster* (*n* = 123*) in Mpumalanga, South Africa, 2014–2020. *Data on reproductive state was only available for 123 individuals.

The most abundant species in 4 of the higher taxa (excluding chiggers) were more abundant and prevalent on male compared to female rodents (Supplementary Table 1). The results of model selection and averaging are presented in Tables 5 and 6. None of the infestation parameters for any of the ectoparasite taxa was significantly associated with host body size. The number of flea and mite individuals were significantly related to host sex and the interaction between host sex and reproductive state (Table 5), with male hosts harbouring more flea and mite individuals than female hosts (Fig. 2A and 2B). Furthermore, the effect of the interaction of host sex and reproductive state on flea and mite numbers in infracommunities was manifested by higher parasite counts on reproductively active males followed by that on non-breeding females (Fig. 2A and 2B; Table 5). In addition, mite species richness was also significantly related to host sex, with a larger number of mite species occurring on males compared to females (Fig. 2C; Table 6).

**Table 5.** Summary of model-averaged (conditional average) coefficients for generalized linear mixed-effects models with negative binomial distribution on the effect of host sex (SX), reproductive state (RS) and habitat type (HBT) on the epifaunistic taxon abundance belonging to different higher taxa on *Gerbilliscus leucogaster* (*n* = 123^a^)

Parasite Taxon	Explanatory variables	Estimate ± s.e.	95% CI	*z*	*R*^2^c	*R*^2^m
Taxon abundance						
Fleas	SX	0.10 ± 0.05*	0.005–0.20	2.05	0.42	0.13
	RS × SX	−0.11 ± 0.04*	−0.19 to −0.02	2.55		
Ticks	HBT	0.22 ± 0.08**	0.06–0.37	2.67	0.61	0.18
Mites	SX	0.01 ± 0.01*	0.001–0.02	2.29	0.77	0.05
	RS × SX	−0.01 ± 0.00**	−0.02 to −0.003	2.67		

The random factor in all models was year. *R*^2^c and *R*^2^m are – conditional and marginal *R*^2^. Significance of estimates –.

a Data on reproductive state was only available for 123 individuals.

***P < 0.001, **P < 0.01, *P < 0.05.

**Table 6.** Summary of model-averaged (conditional average) coefficients for generalized linear mixed-effects models with poisson distribution on the effect of host sex (SX) and habitat type (HBT) on the epifaunistic taxon richness belonging to different higher taxa on *Gerbilliscus leucogaster* (*n* = 123^a^)

Parasite Taxon	Explanatory variables	Estimate ± s.e.	95% CI	*z*	*R*^2^c	*R*^2^m
Taxon richness						
Ticks	HBT	0.84 ± 0.35*	0.15–1.52	2.38	0.27	0.15
Mites	SX	0.09 ± 0.04*	0.009–0.18	2.18	0.59	0.02

The random factor in all models was year. *R*^2^c and *R*^2^m are – conditional and marginal *R*^2^. Significance of estimates – ***P < 0.001, **P < 0.01, *P < 0.05.

a Data on reproductive state was only available for 123 individuals.

**Figure 3.**
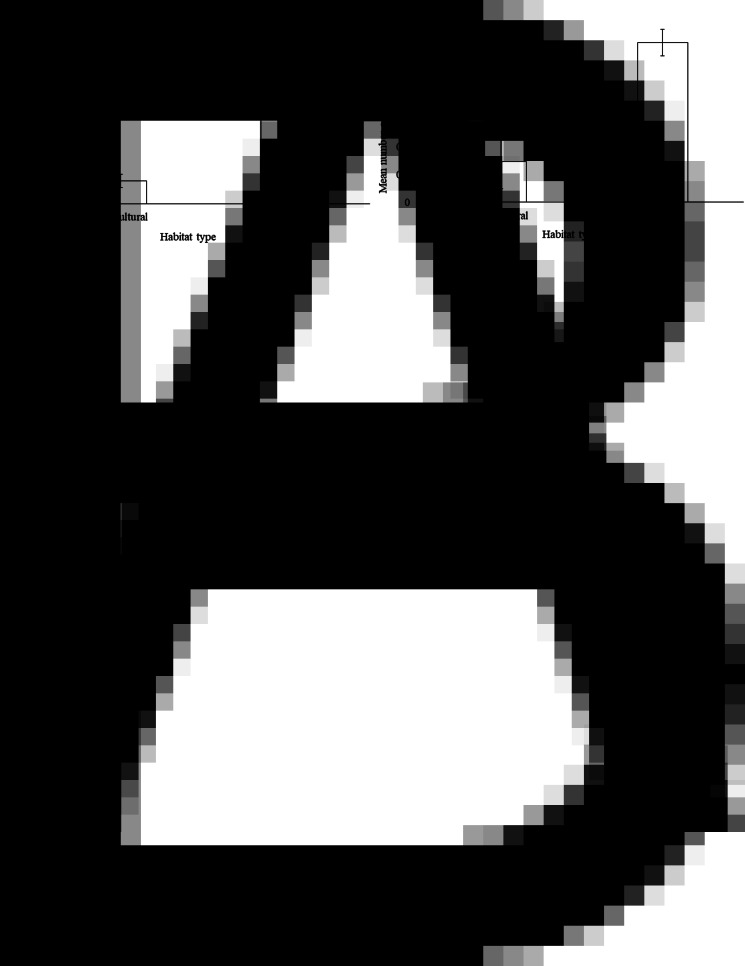
Mean number of: (A) tick individuals (±s.e.) and (B) tick species (±s.e.) per habitat type for *Gerbilliscus leucogaster* (*n* = 127) in Mpumalanga, South Africa, 2014–2020.

Overall, a higher number of epifaunistic individuals were recorded on *G. leucogaster* in natural than agricultural habitats (3984 *vs* 2244, respectively) (Supplementary Table 2). However, a significant relationship was only recorded for ticks with more tick individuals (overall mean: 2.31 ± 0.66 *vs* 0.34 ± 0.16, respectively) and species (overall 5 *vs* 3 species, respectively) collected from rodents captured in natural as compared to agricultural habitats (Fig. 3A and 3B; Tables 5 and 6). The overall prevalence was also higher on *G. leucogaster* that occur in natural (48.53%) compared to agricultural (13.79%) habitat type (Supplementary Table 2).

## Discussion

### Epifaunistic diversity

The flea *X. frayi* was the most common species, which supports a close association between *X. frayi* and *G. leucogaster* as reported by Segerman (1995) and Braack *et al*. (1996). The study area in the present study falls within the known distributional range of *X. frayi*, which spans the eastern and north-eastern Savanna bushveld areas of South Africa (Segerman, 1995). The low occurrence of *X. brasiliensis* may be due to the fact that other rodent species (e.g. *Rattus* spp. and *Mastomys* spp.) represent principal hosts for this flea (Segerman, 1995; Braack *et al*., 1996). The presence of *X. bechuanae* on *G. leucogaster* in the present study could be accidental because (a) *X. bechuanae* is reported as host-specific to the pouched mouse (*Saccostomus campestris*) (Segerman, 1995) co-occurring with *G. leucogaster* and (b) only 2 individuals of *X. bechuanae* were recorded. In addition, a similar pattern was previously recorded for *X. bechuanae* on *G. leucogaster* and *S. campestris* in Namibia (Shihepo *et al*., 2008).

The occurrence of 2 louse species, *H. biseriata* and *P. biseriata*, supports earlier findings (Ledger, 1980; Braack *et al*., 1996). *Polyplax biseriata* was the most prevalent of the 2 species, and only 4 *H. biseriata* individuals were recorded. The dominance of *P. biseriata* is supported by Braack *et al*. (1996). Unfortunately, Braack *et al*. (1996) did not provide differential prevalence values for the 2 louse species, but rather an overall prevalence of 71.70%, which is comparable to the 76.38% recorded in our study.

The occurrence of *H. truncatum* on *G. leucogaster* in the present study supports the findings of Braack *et al*. (1996) who recorded the tick species on 20% of *G. leucogaster* at a locality in the same geographic region as the present study. According to Horak *et al*. (2018) the immature stages of *H. truncatum* seem to prefer *G. leucogaster* in addition to some other host species. *Dermacentor rhinocerinus* was the second most prevalent tick. Horak and Cohen (2001) also recorded this tick on *G. leucogaster* in the Mthethomusha Game Reserve in Mpumalanga Province. It is thus possible that *G. leucogaster* is a preferred host of the immature stages of this tick. Morphological stasis of larval and nymph life stages often limits species-level identification for ticks in the genera *Rhipicephalus* and *Haemaphysalis* (see Walker *et al*., 2000 for the genus *Rhipicephalus*).

Mites (including chiggers) represented the majority (23 of the 32 species) of the epifaunistic arthropods on *G. leucogaster*. The 3 most prevalent mite species are parasitic and belong to Laelapidae (*Androlalaps oliffi*, *A. marshalli* and *A. theseus*). Two species (*A. marshalli* and *A. theseus*) were previously recorded on *G. leucogaster* in the Savanna biome (Braack *et al*., 1996). Zumpt (1961) also lists *A. oliffi*, *A. marshalli*, *A. taterae* and *A. theseus* on several *Gerbilliscus* species (including *G. leucogaster*). The dominance of *Androlaelaps* species, compared to *Laelaps* species, on *G. leucogaster* in the present supports earlier findings (Braack *et al*., 1996) and this), gerbil seems to be the main host for *Androlaelaps* mites in southern Africa (Zumpt, 1961; Till, 1963). The predominance of a female-bias of Laelapidae in the present study is in accordance with previous studies on rodents in South Africa (Matthee *et al*., 2007, 2010) and in other regions (Martins-Hatano *et al*., 2002; Gettinger and Gardner, 2005, 2017). *Androlaelaps* and *Laelaps* females are generally found on the hosts' bodies whereas males and immature individuals are frequently in the nest (Radovsky, 1994). However, exceptions do occur, where male and immature life stages are more represented on the host's body; as seen in *Laelaps dearmasi* (Tipton *et al*., 1966) and *A. oliffi* (this study). We provide the first record of *Listrophoroides (Afrolistrophoroides) mastomys* on *G. leucogaster* (4.75% prevalence). *Listrophoroides (A.) mastomys* was previously recorded on the Natal multimammate mouse (*Mastomys natalensis*) in north and west Africa (e.g. Rwanda, Uganda and Ivory Coast) (Fain, 1972; Dusbabek, 1983). Species in the fur mite genus *Listrophoroides* are associated with rodents, shrews and primates and are globally distributed (Fain and Bochkov, 2004). The presence of another fur mite, the myobiid *Austromyobia forcipifer* on *G. leucogaster* in our study is not surprising as mites in this genus are known exclusively from murid rodents (Bochkov, 2009). Members of the Myobidae are morphologically specialized to attach themselves firmly to the fur and hair of mammals (Wall and Shearer, 2001; Herrera-Mares *et al*., 2021). The macronyssid mite, *Ornithonyssus bacoti* is a bloodsucking ectoparasite that only attaches to the host (birds and mammals) during feeding (Wall and Shearer, 2001). Although the presence of this species on *G. leucogaster* is the first record, it has been recorded on the four-striped mouse in the Western Cape Province of South Africa (Matthee *et al*., 2007). Interestingly, although *A. forcipifer* and *O. bacoti* were recorded in low abundance and prevalence in the present study, they were both more common in the agricultural habitat type. The 4 predatory mite species that were recorded represent 4 families: Pachylaelapidae (*Pachylaelaps* sp.), Uropodidae, Cheyletidae (*C. zumpti*) and Acaroidae. The Pachylaelapidae are predators of micro-fauna (arthropods and soil-dwelling nematodes) in litter, humus, moss and are found in the nests of mammals, birds and insects (Lindquist *et al*., 2009). Uropodidae are found in highly organic, insular deposits of manure and compost where they feed on bacteria, fungi, ants, nematodes and other mites (Lindquist *et al*., 2009). Approximately 78% of cheyletid species are predators, the remaining species are permanent parasites of mammals and birds. *Cheyletus zumpti* was previously recorded in the nests of rodents at various localities in South Africa and tropical African countries (Rwanda, Nigeria, Angola and the Democratic Republic of the Congo) (Fain and Bochkov, 2001). A single specimen was previously found on *G. leucogaster* in Skukuza in the Kruger National Park (Zumpt, 1961). Predacious individuals occupy a wide variety of habitats including plant and soil-litter and are mostly associated with nests of vertebrates or stored grains (Hughes, 1976; Bochkov and OConnor, 2004). The deutonymphs of the Acaroidae recorded in this study, attach themselves to insects or fur of animals, and use them as transport vehicles between habitats (also known as phoresy). Members of the Acaroidae are mainly fungivorous or saprophytic (Lindquist *et al*., 2009).

The chigger, *S. lumsdeni*, is known from the Savanna biome where it was recorded on tree squirrels (*Paraxerus cepapi*) (Zumpt, 1961; Skinner and Chimimba, 2005; Stekolnikov, 2018) and the pouched mouse (Matthee *et al*., 2020). *Gahrliepia nana* is known to parasitize the common mole rat (*Cryptomys hottentotus*), the lesser leaf-nose bat (*Hipposideros caffer*) and the Namaqua rock mouse in the Grassland biome in the central-eastern and eastern region of South Africa (Gauteng and Kwa-Zulu Natal) (Zumpt, 1961; Stekolnikov, 2018; Matthee *et al*., 2020; Stevens *et al*., 2022). *Schoutedenichia morosi* was previously recorded on the Cape gerbil (*Gerbilliscus afra*) and vlei rat (*Otomys irroratus*) in the south-eastern Grassland biome of Lesotho (Zumpt, 1961; Stekolnikov, 2018). Although *S. dutoiti* was described by Zumpt (1961) on the South African pouched mouse in the south-eastern part of South Africa, its presence on *G. leucogaster* has earlier been reported by Matthee *et al*. (2020) in the same locality. *Microtrombicula mastomyia* is known from Central and West Africa where it has a broad host range. Its presence in South Africa was marked as a new country locality by Matthee *et al*. (2020) but it was also reported on the Namaqua rock mouse in the Savanna biome by Stevens *et al*. (2022). The remaining chigger species, *A. ueckermanni*, *S. horaki* and *T. walkerae*, were recently described as new (Stekolnikov and Matthee, 2019). Stekolnikov and Matthee (2019) noted that *Trombicula walkerae* represented the first record of the genus *Trombicula sensu stricto* on the African continent. The *Ascoschoengastia* genus is known from 4 species in Africa. However, the recently described *A. ueckermanni* represents the first record for this genus in South Africa where it has been recorded on *Mastomys* sp. and the Tete veld rat (*Aethomys ineptus*). The genus *Schoutedenichia* is well represented in Africa (Stekolnikov, 2018) and the recently described species, *S. horaki*, has been recorded on *Mastomys* sp. and the pouched mouse.

In this study, the pinna was one of the preferred parasitopes for chiggers. This parasitope was also recorded for a *Leptotrombidium* species on the white-footed mouse (*Peromyscus leucopus*) in northern Michigan (Wrenn, 1974). Additionally, Goff (1979) noted that 96% of *Guntheria omega* were associated with the ear fringe of rodents in Papua New Guinea. In South Africa, this parasitope was previously recorded for chiggers on the Namaqua rock mouse in the Savanna (Fagir *et al*., 2014; Stevens *et al*., 2022) and the Grassland biome (Stevens *et al*., 2022). Here, we found that the tail base was another preferred parasitope. The tail base and perineum of the host were also previously recorded for chiggers on rodents in South Africa (Barnard *et al*., 2015; Stevens *et al*., 2022).

### Effects of host- and habitat-associated factors

Adult males (especially reproductively active) harboured significantly higher flea and mite counts. This pattern is supported by previous studies on ectoparasites associated with rodents in South Africa (Matthee *et al*., 2010; Archer *et al*., 2014; Fagir *et al*., 2015) and elsewhere (Kowalski *et al*., 2015; Hamidi and Bueno-Marí, 2021). As mentioned above, male biased ectoparasite infestation can be a result of several, not mutually exclusive, factors. Among them, sexual size dimorphism cannot explain sexual differences in ectoparasite infestation of *G. leucogaster* because males and females of this species are similar in size, as was also found in our study (Skinner and Chimimba, 2005). Consequently, these differences might be due to other mechanisms. For example, behavioural activities such as grooming and vagility (Krasnov *et al*., 2012; Akinyi *et al*., 2013). Indeed, Lötter and Pillay (2012) reported that female *G. leucogaster* groom more frequently than males. Given that grooming is an effective method to reduce ectoparasite infestations (Hawlena *et al*., 2007, 2008), this may explain lower flea and mite counts and fewer mite species on females (Hart *et al*., 1992; Mooring *et al*., 2004). Regarding vagility, our sampling was mainly carried out during the breeding season (September and October) of *G. leucogaster*. It is thus possible that reproductively active males roamed more widely than females (Wang *et al*., 2011; Gromov, 2012). Burdelov *et al*. (2007) demonstrated that starving fleas are positively phototactic and will therefore cluster at the openings of abandoned burrows and wait for a potential host (Darskaya and Besedina, 1961). More frequent roaming and larger home ranges by reproductively active males may result in higher visitation rates at burrows of other rodents, where they may encounter fleas and mites (Krasnov and Matthee, 2010). Although elevated testosterone levels during the breeding season may be another important contributing factor to male-biased infestations (Zuk and McKean, 1996; Hughes and Randolph, 2001; Ezenwa *et al*., 2012), there is not consistent support for the association between high testosterone levels and parasite infestations (Grear *et al*., 2009; O'Brien *et al*., 2018).

In the present study, hosts captured in the natural compared to the agricultural habitat harboured more ticks. Several ixodid tick species require multiple host species and a favourable external environment, such as vegetation, to complete their life cycle (Cupp, 1991; Horak *et al*., 2018). It is therefore not surprising that studies have reported a significant relationship between habitat type and tick occurrence (Gray, 1998; Jaenson *et al*., 2009; Ledger *et al*., 2019). The vegetation structure that is associated with a particular habitat type can have direct and indirect effects on ticks. Firstly, vegetation structure can directly affect the microclimatic conditions to which free-living tick life stages are exposed (Schulze and Jordan, 2005; Tack *et al*., 2012; Ledger *et al*., 2019). For example, canopies of woody plants alter the microclimate beneath and around them by intercepting precipitation and by shading, which increases soil moisture (Breshears *et al*., 1998; Potts *et al*., 2010; Lozano-Parra *et al*., 2018). In addition, a layer of vegetation and leaf litter can insulate the soil and buffer it against extreme heat and cold temperatures (Pierson and Wight, 1991; Breshears *et al*., 1997). Tick development and survival in the external environment is therefore facilitated in more sheltered habitats with a permanent vegetation layer and a more stable microclimate (Pfäffle *et al*., 2013; Paul *et al*., 2016). Tick genera recorded in the present study are 2- and 3-host ticks (i.e. those that need to find 2 or 3 different hosts, respectively, to complete their life cycle) (Horak *et al*., 2018). Favourable microclimatic conditions seem to be particularly important for these taxa as their larval and nymphal life stages quest for hosts from the soil surface or from grass tufts (Horak and Cohen, 2001; Gallivan *et al*., 2011). In addition, shrub and grass cover is important for ticks to quest and search for a host (Ledger *et al*., 2019; Mathews-Martin *et al*., 2020). Apart from *Hy. truncatum* nymphs, all the nymphs from the tick taxa are dependent on vegetation to quest and find a host (Horak *et al*., 2018). In the present study, the pristine natural habitat had a higher proportion of grass (80 and 79%, respectively) than the agricultural habitat type (70 and 62%, respectively) during August 2014 and January 2015 (S. Matthee unpublished data). It is therefore possible that natural habitats provide a conducive microclimate and physical structures that facilitate tick development and survival (Dube *et al*., 2018; Shilereyo *et al*., 2022). Lastly, in the present study the natural habitat supports a larger diversity of vertebrate hosts and a larger diversity of small and large-bodied vertebrate families (e.g. Bovidae, Canidae, Giraffidae and Rhinocerotidae) (Du Toit, 2003) of which the latter act as natural hosts for the adult life stages (Horak *et al*., 2018). This contrasts with the agricultural habitat type that comprises small crop fields that generally harbour rodents and are infrequently visited by dogs, cattle and goats (personal observation). In addition, habitat types with a higher proportion of larger bodied vertebrate hosts have a higher abundance of ticks (Horak *et al*., 2022) and more adult tick life stages (Esser *et al*., 2016).

This study represents the first systematic long-term assessment of the ectoparasite species associated with *G. leucogaster*. We conclude that *G. leucogaster* is host to a large diversity of epifaunistic species of which mites represent a significant proportion. The relationships recorded between ectoparasite infestations, and the host and habitat factors were life history specific. In particular, the level of infestation by ectoparasites closely associated with the host (fleas and mites) was affected by host-associated factors, while infestation by ectoparasites that spend most of their life in the external environment (ticks) was affected by habitat type. Although the study was limited to local conditions, it provides a valuable baseline for future broader scale studies on *G. leucogaster* in South and southern Africa.

## Data Availability

All data generated or analysed during this study are included in this published article. The datasets used and/or analysed are available from the corresponding author upon reasonable request.

## Supplementary material

For supplementary material accompanying this paper visit https://doi.org/10.1017/S0031182023000562. click here to view supplementary material

## References

[ref1] Akinyi MY, Tung J, Jeneby M, Patel NB, Altmann J and Alberts SC (2013) Role of grooming in reducing tick load in wild baboons (*Papio cynocephalus*). Animal Behaviour 85, 559–568. 24659824 10.1016/j.anbehav.2012.12.012 PMC3961061

[ref2] Apps P (2012) Smither's Mammals of Southern Africa: A Field Guide, 4th Edn. Cape Town, South Africa: Penguin Random House South Africa.

[ref3] Archer EK, Bennett NC, Ueckermann EA and Lutermann H (2014) Ectoparasite burdens of the common mole-rat (*Cryptomys hottentotus hottentotus*) from the Cape Provinces of South Africa. Journal of Parasitology 100, 79–84. 24171714 10.1645/13-270.1

[ref4] Barnard K, Krasnov BR, Goff L and Matthee S (2015) Infracommunity dynamics of chiggers (Trombiculidae) parasitic on a rodent. Parasitology 142, 1605–1611. 26303191 10.1017/S0031182015001110

[ref5] Bartoń K (2018) Mu-MIn: Multi-model inference. Available at https://CRAN.R-project.org/package=MuMIn (accessed 10 January 2022).

[ref6] Bastos AD, Nair D, Taylor PJ, Brettschneider H, Kirsten F, Mostert E, von Maltitz E, Lamb JM, van Hooft P, Belmain SR, Contrafatto G, Downs S and Chimimba CT (2011) Genetic monitoring detects an overlooked cryptic species and reveals the diversity and distribution of three invasive *Rattus* congeners in South Africa. BMC Genetics 12, 26. 21324204 10.1186/1471-2156-12-26 PMC3055845

[ref7] Bates DM, Maechler M, Bolker BM and Walker SC (2015) Fitting linear mixed models with lme4. Journal of Statistical Software 67, 1–7.

[ref8] Bergallo HG and Magnusson WE (2004) Factors affecting the use of space by two rodent species in Brazilian Atlantic forest. Mammalia 68, 121–132.

[ref9] Berrian AM, van Rooyen J, Martínez-López B, Knobel D, Simpson GJG, Wilkes MS and Conrad PA (2016) One Health profile of a community at the wildlife-domestic animal interface, Mpumalanga, South Africa. Preventive Veterinary Medicine 130, 119–128. 27435655 10.1016/j.prevetmed.2016.06.007

[ref10] Bochkov AV (2009) Mites of the family myobiidae (Acari: Prostigmata) parasitizing rodents of the former USSR. Acarina 17, 109–169.

[ref11] Bochkov AV and OConnor BM (2004) Phylogeny, taxonomy and biology of mites of the genera *Chelacheles* and *Neochelacheles* (Acari: Cheyletidae). Invertebrate Systematics 18, 547–592.

[ref12] Bordes F, Blumstein DT and Morand S (2007) Rodent sociality and parasite diversity. Biology Letters 3, 692–694. 17925270 10.1098/rsbl.2007.0393 PMC2391225

[ref13] Braack LEO, Horak IG, Jordaan LC, Segerman J and Louw JP (1996) The comparative host status of red veld rats (*Aethomys chrysophilus*) and Bushveld gerbils (*Tatera leucogaster*) for epifaunal arthropods in the southern Kruger National Park, South Africa. Onderstepoort Journal of Veterinary Research 63, 149–158. 8856764

[ref14] Breshears DD, Rich PM, Barnes FJ and Campbell K (1997) Overstory-imposed heterogeneity in solar radiation and soil moisture in a Semi-arid woodland. Ecological Applications 7, 1201–1215.

[ref15] Breshears DD, Nyhan JW, Heil CE and Wilcox BP (1998) Effects of woody plants on microclimate in a semiarid woodland: soil temperature and evaporation in canopy and intercanopy patches. International Journal of Plant Sciences 159, 1010–1017.

[ref16] Burdelov SA, Leiderman M, Khokhlova IS, Krasnov BR and Degen AA (2007) Locomotor response to light and surface angle in three species of desert fleas. Parasitology Research 100, 973–982. 17136383 10.1007/s00436-006-0383-9

[ref17] Butler RA, Trout Fryxell RT, Houston AE, Bowers EK, Paulsen D, Coons LB and Kennedy ML (2020) Small-mammal characteristics affect tick communities in southwestern Tennessee (USA). International Journal for Parasitology: Parasites and Wildlife 12, 150–154. 32547921 10.1016/j.ijppaw.2020.05.012 PMC7284121

[ref18] Cameron G (2000) Community ecology of subterranean rodents. In Lacey E, Patton J and Cameron G (eds), Life Underground: The Biology of Subterranean Rodents. Illinois, USA: University of Chicago Press, pp. 227–256.

[ref19] Choate TS (1972) Behavioural studies on some Rhodesian rodents. Zoologica Africana 7, 103–118.

[ref20] Christe P, Arlettaz R and Vogel P (2000) Variation in intensity of a parasitic mite (*Spinturnix myoti*) in relation to the reproductive cycle and immunocompetence of its bat host (*Myotis myotis*). Ecology Letters 3, 207–212.

[ref21] Cupp EW (1991) Biology of Ticks. The Veterinary clinics of North America. Small animal practice 21, 1–26. 10.1016/s0195-5616(91)50001-2 2014614

[ref22] Darskaya N and Besedina K (1961) On the possibility of flea feeding on reptiles. Scientific-Research Anti-Plague Institute of the Caucasus and Trans-Caucasus 5, 33–39.

[ref23] De Graaff G (1981) The Rodents of Southern Africa. Durban, South Africa: Butterworth and Co, p. 267.

[ref24] Dube WC, Hund AK, Turbek SP and Safran RJ (2018) Microclimate and host body condition influence mite population growth in a wild bird-ectoparasite system. International Journal for Parasitology: Parasites and Wildlife 7, 301–308. 30128287 10.1016/j.ijppaw.2018.07.007 PMC6097460

[ref25] Durden LA and Musser GG (1994) The Sucking Lice (Insecta, Anoplura) of the World- A Taxonomic Checklist with Records of Mammalian Hosts and Geographical Distributions No. 218. New York, USA: Bulletin of the American Museum of Natural History.

[ref26] Dusbabek F (1983) Some parasitic Prostigmata and Astigmata (Acarina) of small mammals in Toro Game Reserve, Uganda. Folia parasitologica 30, 47–55. 7106656

[ref27] Du Toit JT (2003) Large herbivores and savanna heterogeneity. In du Toit JT, Rogers KH and Biggs HC (eds), The Kruger Experience: Ecology And Management Of Savanna Heterogeneity. Washington, DC: Island Press, pp. 292–309.

[ref28] Esser HJ, Foley JE, Bongers F, Herre EA, Miller MJ, Prins HHT and Jansen PA (2016) Host body size and the diversity of tick assemblages on Neotropical vertebrates. International Journal for Parasitology: Parasites and Wildlife 5, 295–304. 27812506 10.1016/j.ijppaw.2016.10.001 PMC5078680

[ref29] Ezenwa VO, Stefan Ekernas L and Creel S (2012) Unravelling complex associations between testosterone and parasite infection in the wild. Functional Ecology 26, 123–133.

[ref30] Fagir DM, Ueckermann EA, Horak IG, Bennett NC and Lutermann H (2014) The Namaqua rock mouse (*Micaelamys namaquensis*) as a potential reservoir and host of arthropod vectors of diseases of medical and veterinary importance in South Africa. Parasites and Vectors 7, 366. 25127720 10.1186/1756-3305-7-366 PMC4141090

[ref31] Fagir DM, Horak IG, Ueckermann EA, Bennett NC and Lutermann H (2015) Ectoparasite diversity in the Eastern Rock Sengis (*Elephantulus myurus*): the effect of seasonality and host sex. African Zoology 50, 109–117.

[ref32] Fagir DM, Bennett NC, Ueckermann EA, Howard A and Hart DW (2021) Ectoparasitic community of the Mahali mole-rat, *Cryptomys hottentotus mahali*: potential host for vectors of medical importance in South Africa. Parasites and Vectors 14, 24. 33407807 10.1186/s13071-020-04537-w PMC7788776

[ref33] Fain A (1972) Les Listrophorides en Afrique au Sud du Sahara (Acarina: Sarcoptiformes). Annales Musee royal de I'Afrique Centrale. (Zoologie) 197, 1–200.

[ref34] Fain A and Bochkov AV (2001) A review of the genus *Cheyletus* Latreille, 1776 (Acari: Cheyletidae). Bulletin de L'institut Royal des Sciences Naturelles de Belgique, Entomologie 71, 83–114.

[ref35] Fain A and Bochkov AV (2004) *Listrophoroides* (*Afrolistrophoroides*) *prionomys* sp. n.(Acari, Atopomelidae) parasitic on *Prionomys batesi* (Rodentia, Dendromurinae) from Republique Centrafricaine. Journal of Afrotropical Zoology 1, 5–8.

[ref36] Flores-Peredo R, Sánchez-Velásquez LR, Galindo-González J and Morales-Mávil JE (2011) Post-dispersed pine seed removal and its effect on seedling establishment in a Mexican Temperate Forest. Plant Ecology 212, 1037–1046.

[ref37] Froeschke G and Matthee S (2014) Landscape characteristics influence helminth infestations in a peri-domestic rodent- Implications for possible zoonotic disease. Parasites and Vectors 7, 393. 25159989 10.1186/1756-3305-7-393 PMC4158073

[ref38] Froeschke G, van der Mescht L, McGeoch M and Matthee S (2013) Life history strategy influences parasite responses to habitat fragmentation. International Journal for Parasitology 43, 1109–1118. 23954434 10.1016/j.ijpara.2013.07.003

[ref39] Galiano D, Kubiak BB, Overbeck GE and de Freitas TRO (2014) Effects of rodents on plant cover, soil hardness, and soil nutrient content: a case study on tuco-tucos (*Ctenomys minutus*). Acta Theriologica 59, 583–587.

[ref40] Gallivan GJ, Spickett A, Heyne H, Spickett AM and Horak IG (2011) The dynamics of questing ticks collected for 164 consecutive months off the vegetation of two landscape zones in the Kruger National Park (1988–2002). Part III. The less commonly collected species. Onderstepoort Journal of Veterinary Research 78, 27–35. 10.4102/ojvr.v78i1.41 23327206

[ref41] Ganem G and Bennett NC (2004) Tolerance to unfamiliar conspecifics varies with social organization in female African mole-rats. Physiology and Behavior 82, 555–562. 15276822 10.1016/j.physbeh.2004.05.002

[ref42] Gettinger D and Gardner SL (2005) Bolivian ectoparasites: a new species of laelapine mite (Acari: Parasitiformes, Laelapidae) from the rodent *Neacomys spinosus*. Journal of Parasitology 91, 49–52. 15856871 10.1645/GE-3371

[ref43] Gettinger D and Gardner SL (2017) Ectoparasitic mites of the genus *Gigantolaelaps* (Acari: Mesostigmata: Laelapidae) associated with small mammals of the genus *Nephelomys* (Rodentia: Sigmodontinae), including two new species from Peru. Acarologia 54, 755–763.

[ref44] Gleason ED, Fuxjager MJ, Oyegbile TO and Marler CA (2009) Testosterone release and social context: when it occurs and why. Frontiers in Neuroendocrinology 30, 460–469. 19422843 10.1016/j.yfrne.2009.04.009

[ref45] Goff ML (1979) Host exploitation by chiggers (Acari: Trombiculidae) infesting Papua New Guinea land mammals. Pacific Insects 20, 321–353.

[ref46] Gray JS (1998) The ecology of ticks transmitting *Lyme borreliosis*. Experimental and Applied Acarology 22, 249–258.

[ref47] Grear DA, Perkins SE and Hudson PJ (2009) Does elevated testosterone result in increased exposure and transmission of parasites? Ecology Letters 12, 528–537. 19392718 10.1111/j.1461-0248.2009.01306.x

[ref48] Gromov VS (2012) Rodents and space: what behavior do we study under semi-natural and laboratory conditions? In Triunveri A and Scalise D (eds), Rodents: Habitat Pathology and Environmental Impact. New York, USA: Nova Science Publishers Inc, pp. 43–59.

[ref49] Hamidi K and Bueno-Marí R (2021) Host-ectoparasite associations; the role of host traits, season and habitat on parasitism interactions of the rodents of northeastern Iran. Journal of Asia-Pacific Entomology 24, 308–319.

[ref50] Hart BL, Hart LA, Mooring MS and Olubayo R (1992) Biological basis of grooming behaviour in antelope: the body-size, vigilance and habitat principles. Animal Behaviour 44, 615–631.

[ref51] Hawlena H, Bashary D, Abramsky Z and Krasnov BR (2007) Benefits, costs and constraints of anti-parasitic grooming in adult and juvenile rodents. Ethology 113, 394–402.

[ref52] Hawlena H, Bashary D, Abramsky Z, Khokhlova IS and Krasnov BR (2008) Programmed versus stimulus-driven antiparasitic grooming in a desert rodent. Behavioral Ecology 19, 929–935.

[ref53] He Y, D'Odorico P, De Wekker SFJ, Fuentes JD and Litvak M (2010) On the impact of shrub encroachment on microclimate conditions in the northern Chihuahuan desert. Journal of Geophysical Research Atmospheres 115, D21120.

[ref54] Herrera-Mares A, Guzman-Cornejo C and Morales-Malacara JB (2021) The myobiid mites (Acariformes, Eleutherengona, Myobiidae) from Mexico: hosts, distribution and identification key for the genera and species. Systematic and Applied Acarology 26, 724–748.

[ref55] Herrin CS and Tipton VJ (1975) Spinturnicid mites of Venezuela (Acarina: Spinturnicidae). Brigham Young University Science Bulletin, Biological Series 20, 1–72.

[ref56] Herrmann C and Gern L (2010) Survival of *Ixodes ricinus* (Acari: Ixodidae) under challenging conditions of temperature and humidity is influenced by *Borrelia burgdorferi* sensu lato infection. Journal of Medical Entomology 47, 1196–1204. 21175072 10.1603/me10111

[ref57] Hopla CE, Durden LA and Keirans JE (1994) Ectoparasites and classification. Revue scientifique et technique (International Office of Epizootics) 13, 985–1017. 7711316 10.20506/rst.13.4.815

[ref58] Horak IG and Cohen M (2001) Hosts of the immature stages of the rhinoceros tick, *Dermacentor rhinocerinus* (Acari, Ixodidae). Onderstepoort Journal of Veterinary Research 68, 75–77. 11403434

[ref59] Horak IG, Heyne H, Williams R, Gallivan GJ, Spickett AM, Bezuidenhout JD and Estrada-Peña A (2018) The Ixodid Ticks (Acari: Ixodidae) of Southern Africa. Scotland, UK: Springer.

[ref60] Horak IG, Junker K and Krasnov BR (2022) Similarity in ixodid tick communities harboured by wildlife and livestock in the Albany thicket biome of South Africa. Parasitology 149, 667–674. 35115071 10.1017/S0031182022000129 PMC11010466

[ref61] Hughes AM (1976) The mites of stored food and houses. Technical Bulletin – Ministry of Agriculture, Fisheries and Food 9, 1–400.

[ref62] Hughes VL and Randolph SE (2001) Testosterone increases the transmission potential of tick-borne parasites. Parasitology 123, 365–371. 11676368 10.1017/s0031182001008599

[ref63] Jaenson TGT, Eisen L, Comstedt P, Mejlon HA, Lindgren E, BergstrÖm S and Olsen B (2009) Risk indicators for the tick *Ixodes ricinus* and *Borrelia burgdorferi* sensu lato in Sweden. Medical and Veterinary Entomology 23, 226–237. 19712153 10.1111/j.1365-2915.2009.00813.x

[ref64] Jucker T, Hardwick SR, Both S, Elias DMO, Ewers RM, Milodowski DT, Swinfield T and Coomes DA (2018) Canopy structure and topography jointly constrain the microclimate of human-modified tropical landscapes. Global Change Biology 24, 5243–5258. 30246358 10.1111/gcb.14415

[ref65] Klein SL (2004) Hormonal and immunological mechanisms mediating sex differences in parasite infection. Parasite Immunology 26, 247–264. 15541029 10.1111/j.0141-9838.2004.00710.x

[ref66] Kołodziej-Sobocińska M (2019) Factors affecting the spread of parasites in populations of wild European terrestrial mammals. Mammal Research 64, 301–318.

[ref67] Kowalski K, Bogdziewicz M, Eichert U and Rychlik L (2015) Sex differences in flea infections among rodent hosts: is there a male bias? Parasitology Research 114, 337–341. 25410932 10.1007/s00436-014-4231-z PMC4281372

[ref68] Krasnov BR and Matthee S (2010) Spatial variation in gender-biased parasitism: host-related, parasite-related and environment-related effects. Parasitology 137, 1527–1536. 20550754 10.1017/S0031182010000454

[ref69] Krasnov BR, Khokhlova IS, Fielden LJ and Burdelova NV (2001) Effect of air temperature and humidity on the survival of pre-imaginal stages of two flea species (Siphonaptera: Pulicidae). Journal of Medical Entomology 38, 629–637. 11580034 10.1603/0022-2585-38.5.629

[ref70] Krasnov BR, Khokhlova I and Shenbrot G (2002) The effect of host density on ectoparasite distribution: an example of a rodent parasitized by fleas. Ecology 83, 164–175.

[ref71] Krasnov BR, Morand S, Hawlena H, Khokhlova IS and Shenbrot GI (2005) Sex-biased parasitism, seasonality and sexual size dimorphism in desert rodents. Oecologia 146, 209–217. 16025350 10.1007/s00442-005-0189-y

[ref72] Krasnov BR, Matthee S, Lareschi M, Korallo-Vinarskaya NP and Vinarski MV (2010) Co-occurrence of ectoparasites on rodent hosts: null model analyses of data from three continents. Oikos 119, 120–128.

[ref73] Krasnov BR, Bordes F, Khokhlova IS and Morand S (2012) Gender-biased parasitism in small mammals: patterns, mechanisms, consequences. Mammalia 76, 1–13.

[ref74] Ledger JA (1980) The arthropod parasites of vertebrates in Africa south of the Sahara. Volume IV. Phthiraptera (Insecta). Johannesburg, South African Institute for Medical Research.

[ref75] Ledger KJ, Keenan RM, Sayler KA and Wisely SM (2019) Multi-scale patterns of tick occupancy and abundance across an agricultural landscape in Southern Africa. PLoS ONE 14, e0222879. 31539412 10.1371/journal.pone.0222879 PMC6754170

[ref76] Lightfoot JT (2008) Sex hormones’ regulation of rodent physical activity: a review. International Journal of Biological Sciences 4, 126–132. 18449357 10.7150/ijbs.4.126 PMC2359866

[ref77] Lindenfors P, Nunn CL, Jones KE, Cunningham AA, Sechrest W and Gittleman JL (2007) Parasite species richness in carnivores: effects of host body mass, latitude, geographical range and population density. Global Ecology and Biogeography 16, 496–509.

[ref78] Lindquist EE, Krantz GW and Walter DE (2009) Classification. In Krantz GW and Walter D (eds), A Manual of Acarology, 3rd Edn. Lubbock, USA: Texas Tech University Press, pp. 97–103.

[ref79] Lorch D, Fisher DO and Spratt DM (2007) Variation in ectoparasite infestation on the brown antechinus, *Antechinus stuartii*, with regard to host, habitat and environmental parameters. Australian Journal of Zoology 55, 169–176.

[ref80] Lötter TK (2010) *Sociality and reproductive biology of the bushveld gerbil* Gerbilliscus leucogaster (PhD thesis). University of the Witwatersrand, Johannesburg, South Africa.

[ref81] Lötter TK and Pillay N (2012) Social interactions associated with reproduction in the bushveld gerbil *Gerbilliscus leucogaster*. Acta Theriologica 57, 29–39.

[ref82] Lozano-Parra J, Pulido M, Lozano-Fondón C and Schnabel S (2018) How do soil moisture and vegetation covers influence soil temperature in drylands of Mediterranean regions? Water 10, 1747.

[ref83] Marshall AG (1981) The Ecology of Ectoparasitic Insects. London, UK: Academic Press.

[ref84] Martins-Hatano F, Gettinger D and Bergallo HG (2002) Ecology and host specificity of laelapine mites (Acari: Laelapidae) of small mammals in an Atlantic forest area of Brazil. Journal of Parasitology 88, 36–40. 12053977 10.1645/0022-3395(2002)088[0036:EAHSOL]2.0.CO;2

[ref85] Mathews-Martin L, Namèche M, Vourc'h G, Gasser S, Lebert I, Poux V, Barry S, Bord S, Jachacz J, Chalvet-Monfray K, Bourdoiseau G, Pamies S, Sepúlveda D, Chambon-Rouvier S and René-Martellet M (2020) Questing tick abundance in urban and peri-urban parks in the French city of Lyon. Parasites and Vectors 13, 576. 33183354 10.1186/s13071-020-04451-1 PMC7659073

[ref86] Matthee S and Krasnov BR (2009) Searching for generality in the patterns of parasite abundance and distribution: ectoparasites of a South African rodent, *Rhabdomys pumilio*. International Journal for Parasitology 39, 781–788. 19168068 10.1016/j.ijpara.2008.12.003

[ref87] Matthee S and Ueckermann EA (2008) Ectoparasites of rodents in Southern Africa: a new species of *Androlaelaps* Berlese, 1903 (Acari: Parasitiformes: Laelapidae) from *Rhabdomys pumilio* (Sparrman) (Rodentia: Muridae). Systematic Parasitology 70, 185–190. 18535789 10.1007/s11230-008-9130-1

[ref88] Matthee S and Ueckermann EA (2009) Ectoparasites of rodents in Southern Africa: two new species of *Laelaps* Koch, 1836 (Acari: Laelapidae) ectoparasitic on *Rhabdomys pumilio* (Sparrman) (Rodentia: Muridae). Systematic Parasitology 73, 27–35. 19337857 10.1007/s11230-009-9181-y

[ref89] Matthee S, Horak IG, Beaucournu J, Durden LA, Ueckermann EA and Mcgeoch M A (2007) Epifaunistic arthropod parsites of the four-striped mouse, *Rhabdomys pumilio*, in the Western Cape Province, South Africa. Journal of Parasitology 93, 47–59. 17436941 10.1645/GE-819R2.1

[ref90] Matthee S, McGeoch MA and Krasnov BR (2010) Parasite-specific variation and the extent of male-biased parasitism; an example with a South African rodent and ectoparasitic arthropods. Parasitology 137, 651–660. 19835648 10.1017/S0031182009991338

[ref91] Matthee S, Stekolnikov AA, Van Der Mescht L, Froeschke G and Morand S (2020) The diversity and distribution of chigger mites associated with rodents in the South African Savanna. Parasitology 147, 1038–1047. 32364099 10.1017/S0031182020000748 PMC10317680

[ref92] Meaney M and Stewart J (1979) Environmental factors influencing the affiliative behavior of male and female rats (*Rattus norvegicus*). Animal Learning and Behavior 7, 397–405.

[ref93] Midgley J and Anderson B (2005) Scatterhoarding in Mediterranean shrublands of the SW Cape, South Africa. In Forget PM, Lambert JE, Hulme PE and Vander Wall SB (eds), Seed Fate: Predation, Dispersal and Seedling Establishment. Oxfordshire, UK: CABI Publishing, pp. 197–204.

[ref94] Monadjem A, Taylor PJ, Denys C and Cotterill FPD (2015) Rodents of Sub-Saharan Africa: A Biogeographic and Taxonomic Synthesis. Berlin, Germany: Walter de Gruyter GmbH & Co KG.

[ref95] Moore SL and Wilson K (2002) Parasites as a viability cost of sexual selection in natural populations of mammals. Science (New York, N.Y.) 297, 2015–2018. 12242433 10.1126/science.1074196

[ref96] Mooring MS, Blumstein DT and Stoner CJ (2004) The evolution of parasite-defence grooming in ungulates. Biological Journal of the Linnean Society 81, 17–37.

[ref97] Morand S and Poulin R (1998) Density, body mass and parasite species richness of terrestrial mammals. Evolutionary Ecology 12, 717–727.

[ref98] Morand S, Krasnov BR, Poulin R and Degen AA (2006) Micromammals and macroparasites: who is who and how they interact? In Morand S, Krasnov BR and Poulin R (eds), Micromammals and Macroparasites: From Evolutionary Ecology to Management. Berlin, Germany: Springer, pp. 3–6. doi: 10.1007/978-4-431-36025-4

[ref99] Nakagawa S, Johnson PCD and Schielzeth H (2017) The coefficient of determination *R*^2^ and intra-class correlation coefficient from generalized linear mixed-effects models revisited and expanded. Journal of the Royal Society Interface 14, 20170213. 28904005 10.1098/rsif.2017.0213 PMC5636267

[ref100] Neal BR (1991) Seasonal changes in reproduction and diet of the Bushveld gerbil, *Tatera leucogaster* (Muridae: Rodentia), in Zimbabwe. Z. Säugetierkunde 56, 101–111.

[ref101] Nyirenda VR, Namukonde N, Simwanda M, Phiri D, Murayama Y, Ranagalage M and Milimo K (2020) Rodent assemblages in the mosaic of habitat types in the Zambezian bioregion. Diversity 12, 365.

[ref102] Obiegala A, Arnold L, Pfeffer M, Kiefer M, Kiefer D, Sauter-Louis C and Silaghi C (2021) Host–parasite interactions of rodent hosts and ectoparasite communities from different habitats in Germany. Parasites and Vectors 14, 112. 33596984 10.1186/s13071-021-04615-7 PMC7890891

[ref103] O'Brien KA, Waterman JM, Anderson WG and Bennett NC (2018) Trade-offs between immunity and testosterone in male African ground squirrels. Journal of Experimental Biology 221, jeb177683. 29941613 10.1242/jeb.177683

[ref104] Odhiambo RO, Makundi RH, Leirs H and Verhagen R (2008) Demography, reproductive biology and diet of the bushveld gerbil *Tatera leucogaster* (Rodentia: Gerbillinae) in the Lake Rukwa valley, south-western Tanzania. Integrative Zoology 3, 31–37. 21396049 10.1111/j.1749-4877.2008.00073.x

[ref105] Paramasvaran S, Sani RA, Hassan L, Krishnasamy M, Jeffery J, Oothuman P, Salleh I, Lim KH, Sumarni MG and Santhana RL (2009) Ectoparasite fauna of rodents and shrews from four habitats in Kuala Lumpur and the states of Selangor and Negeri Sembilan, Malaysia and its public health significance. Tropical Biomedicine 26, 303–311. 20237444

[ref106] Patterson JEH and Ruckstuhl KE (2013) Parasite infection and host group size: a meta-analytical review. Parasitology 140, 803–813. 23425516 10.1017/S0031182012002259 PMC3638372

[ref107] Paul REL, Cote M, Le Naour E and Bonnet SI (2016) Environmental factors influencing tick densities over seven years in a French suburban forest. Parasites and Vectors 9, 309. 27234215 10.1186/s13071-016-1591-5 PMC4884405

[ref108] Perrin MR and Swanepoel P (1987) Breeding biology of the bushveld gerbil *Tatera leucogaster* in relation to diet, rainfall and life history theory. South African Journal of Zoology 22, 218–227.

[ref109] Pfäffle M, Littwin N, Muders SV and Petney TN (2013) The ecology of tick-borne diseases. International Journal for Parasitology 43, 1059–1077. 23911308 10.1016/j.ijpara.2013.06.009

[ref110] Pierson FB and Wight JR (1991) Variability of near-surface soil temperature on sagebrush rangeland. Journal of Range Management 44, 491–497.

[ref111] Potts DL, Scott RL, Bayram S and Carbonara J (2010) Woody plants modulate the temporal dynamics of soil moisture in a semi-arid mesquite Savanna. Ecohydrology: Ecosystems, Land and Water Process Interactions, Ecohydrogeomorphology 130, 126–130.

[ref112] Poulin R (2007) Evolutionary Ecology of Parasites: From Individuals to Communities. Princeton, New Jersey: Princeton University Press.

[ref113] Radovsky FJ (1994) The evolution of parasitism and the distribution of some Dermanyssoid mites (Mesostigmata) on vertebrate hosts. In Houck MA (ed.), Mites: Ecological and Evolutionary Analyses of Life-History Patterns. New York, USA: Chapman & Hall, pp. 186–217.

[ref114] R Core Team (2020) R: A Language and Environment for Statistical Computing. Vienna, Austria: R Foundation for Statistical Computing. Available at https://www.R-project.org/.

[ref115] Reichman O (2007) The influence of pocket gophers on the biotic and abiotic environment. In Begal S, Burda H and Schleich C (eds), Subterranean Rodents: News From Underground. Heidelberg, Germany: Springer-Verlag Berlin, pp. 271–286.

[ref116] Scantlebury M, Maher McWilliams M, Marks N, Dick JTA, Edgar H and Lutermann H (2010) Effects of life-history traits on parasite load in grey squirrels. Journal of Zoology 282, 246–255.

[ref117] Schulze TL and Jordan RA (2005) Influence of meso- and microscale habitat structure on focal distribution of sympatric *Ixodes scapularis* and *Amblyomma americanum* (Acari: Ixodidae). Journal of Medical Entomology 42, 285–294. 15962776 10.1093/jmedent/42.3.285

[ref118] Segerman J (1995) Siphonaptera of Southern Africa: Handbook for the Identification of Fleas. Johannesburg, South Africa: Publications of the South African Institute for Medical Research.

[ref119] Shihepo FG, Eiseb S and Cunningham P (2008) Fleas (Insecta: Siphonaptera) associated with small mammals in selected areas in northern Namibia. Journal Namibia Scientific Society 56, 5–23.

[ref120] Shilereyo M, Magige F, Ranke PS, Ogutu JO and Røskaft E (2022) Ectoparasite load of small mammals in the Serengeti ecosystem: effects of land use, season, host species, age, sex and breeding status. Parasitology Research 121, 823–838. 35122139 10.1007/s00436-022-07439-1 PMC8858283

[ref121] Simon NG and Lu S (2006) Androgens and aggression. In Nelson RJ (ed.), Biology of Aggression. New York, USA: Oxford University Press, pp. 211–230.

[ref122] Skinner JD and Chimimba CT (2005) The Mammals of the Southern African Subregion, 3rd Edn., Cape Town, South Africa: Cambridge University Press.

[ref123] Stanko M, Fričová J, Miklisová D, Khokhlova IS and Krasnov BR (2015) Environment-related and host-related factors affecting the occurrence of lice on rodents in Central Europe. Parasitology 142, 938–947. 25651932 10.1017/S0031182015000037

[ref124] Stekolnikov AA (2018) Taxonomy and distribution of African chiggers. European Journal of Taxonomy 395, 1–233.

[ref125] Stekolnikov AA and Matthee S (2019) Six new and one little known species of chigger mites (Acariformes: Trombiculidae) from South Africa. Systematic and Applied Acarology 24, 435–466.

[ref126] Stevens L, Stekolnikov AA, Ueckermann EA, Horak IG and Matthee S (2022) Diversity and distribution of ectoparasite taxa associated with *Micaelamys namaquensis* (Rodentia: Muridae), an opportunistic commensal rodent species in South Africa. Parasitology 149, 1229–1248. 35641880 10.1017/S0031182022000750 PMC10090637

[ref127] Tack W, Madder M, Baeten L, Vanhellemont M, Gruwez R and Verheyen K (2012) Local habitat and landscape affect *Ixodes ricinus* tick abundances in forests on poor, sandy soils. Forest Ecology and Management 265, 30–36.

[ref128] Tew TE and Macdonald DW (1994) Dynamics of space use and male vigour amongst wood mice, *Apodemus sylvaticus*, in the cereal ecosystem. Behavioral Ecology and Sociobiology 34, 337–345.

[ref129] Theiler G (1962) The Ixodoidea parasites of vertebrates in Africa south of the Sahara (Ethiopian region). Project S 9958. Report to the Director of Veterinary Services, Onderstepoort (mimeographed), 260 pp.

[ref130] Till WM (1963) Ethiopian mites of the genus *Androlaelaps* Berlese s. lat. (Acari: Mesostigmata). *Bulletin of the Natural History Museum*. Zoology 10, 1–104.

[ref131] Tipton VJ, Altman RM and Keenan CM (1966) Mites of the subfamily Laelaptinae in Panama (Acarina: Laelaptidae). In Wenzel RL and Tipton VJ (eds), Ectoparasites of Panama. Chicago, USA: Field Museum of Natural History, pp. 23–82.

[ref132] Van der Mescht L, le Roux PC and Matthee S (2013) Remnant fragments within an agricultural matrix enhance conditions for a rodent host and its fleas. Parasitology 140, 368–377. 23101765 10.1017/S0031182012001692

[ref133] Van der Mescht L, Le Roux PC, Matthee CA, Raath MJ and Matthee S (2016) The influence of life history characteristics on flea (Siphonaptera) species distribution models. Parasites and Vectors 9, 178. 27026237 10.1186/s13071-016-1466-9 PMC4812659

[ref134] Vaumourin E, Vourc'h G, Gasqui P and Vayssier-Taussat M (2015) The importance of multiparasitism: examining the consequences of co-infections for human and animal health. Parasites and Vectors 8, 545. 26482351 10.1186/s13071-015-1167-9 PMC4617890

[ref135] Viljoen H, Bennett NC, Ueckermann EA and Lutermann H (2011) The role of host traits, season and group size on parasite burdens in a cooperative mammal. PLoS ONE 6, e27003. 22069481 10.1371/journal.pone.0027003 PMC3206063

[ref136] Von Maltitz EF, Kirsten F and Labuschagne L (2016) Gerbils: ecologically based rodent management in maize. SA Grain March 2016, 22–25.

[ref137] Walker JB, Keirans JE and Horak IG (2000) The Genus Rhipicephalus (Acari, Ixodidae): A Guide to the Brown Ticks of the World. Cambridge, UK: Cambridge University Press.

[ref138] Wall R and Shearer D (2001) The diagnosis and control of ectoparasite infestation. In Wall R and Shearer D (eds), Veterinary Ectoparasites: Biology, Pathology and Control. Oxford, UK: Blackwell Science Ltd, pp. 179–242.

[ref139] Wang Y, Liu W, Wang G, Wan X and Zhong W (2011) Home-range sizes of social groups of Mongolian gerbils *Meriones unguiculatus*. Journal of Arid Environments 75, 132–137.

[ref140] Wester P, Stanway R and Pauw A (2009) Mice pollinate the Pagoda Lily, *Whiteheadia bifolia* (Hyacinthaceae) – first field observations with photographic documentation of rodent pollination in South Africa. South African Journal of Botany 75, 713–719.

[ref141] Wiens D, Rourke JP, Casper BB, Rickart EA, LaPine TR, Paterson CJ and Channing A (1983) Non-flying mammal pollination of Southern African proteas: a non-coevolved system. Annals of the Missouri Botanical Garden 70, 1–31.

[ref142] Wrenn WJ (1974) Notes on the ecology of chiggers (Acarina: Trombiculidae) from Northern Michigan and the Description of a New Species of *Euschoengastia*. Journal of the Kansas Entomological Society 47, 227–238.

[ref143] Yong SK, Jalaludin NH, Brau E, Shamsudin NN and Heo CC (2019) Changes in soil nutrients (ammonia, phosphate and nitrate) associated with rat carcass decomposition under tropical climatic conditions. Soil Research 57, 482–488.

[ref144] Zenuto RR, Vassallo AI and Busch C (2001) A method for studying social and reproductive behaviour of subterranean rodents in captivity. Acta Theriologica 46, 161–170.

[ref145] Zielinski WJ and Vandenbergh JG (1993) Testosterone and competitive ability in male house mice, *Mus musculus*: laboratory and field studies. Animal Behaviour 45, 873–891.

[ref146] Zuk M and McKean KA (1996) Sex differences in parasite infections: patterns and processes. International Journal for Parasitology 26, 1009–1024. 8982783

[ref147] Zumpt F (1961) The Arthropod Parasites of Vertebrates in Africa South of the Sahara (Ethiopian Region). Vol. I (Chelicerata). Johannesburg, South African: Institute for Medical Research.

