# Supplementary material for: Ectoparasites associated with the Bushveld gerbil (Gerbilliscus leucogaster) and the role of the host and habitat in shaping ectoparasite diversity and infestations
Source: Parasitology. 2023 Jun 5;150(9):792–804. doi: 10.1017/S0031182023000562 (PMC10478068; doi:10.1017/S0031182023000562)
Supplement: Supplementary file 1 [file S0031182023000562sup001.docx]

Smith *et al*., Supplementary material

**Table S1.** Mean abundance (±SE) and prevalence of epifaunistic taxa and species recorded on male (*n* = 77) and female (*n* = 50) *Gerbilliscus leucogaster* (*n* = 127) in Mpumalanga, South Africa, 2014–2020.

| **Epifaunistic taxa** | **Male** | | **Female** | |
| --- | --- | --- | --- | --- |
|  | Mean abundance (±SE) | Prevalence (%) | Mean abundance (±SE) | Prevalence (%) |
| All taxa | 49.97 ± 5.37 | 100 | 58.2 ± 8.87 | 96 |
| **Fleas*** | 7.60 ± 0.85 | 83.12 | 2.38 ± 0.60 | 76 |
| *Xenopsylla bechuanae^#^* | 0.01 ± 0.01 | 1.37 | 0.02 ± 0.01 | 2 |
| *X. brasiliensis^#^* | 0.01 ± 0.01 | 1.37 | 0.12 ± 0.02 | 6 |
| *X. frayi^#^* | 4.08 ± 0.44 | 83.56 | 2.24 ± 0.19 | 68 |
| **Lice** | 15.44 ± 2.44 | 77.92 | 12.60 ± 3.19 | 74 |
| *Hoplopleura biseriata* | 0.04 ± 0.02 | 4.11 | 0.02 ± 0.02 | 2 |
| *Polyplax biseriata* | 13.34 ± 2.18 | 75.34 | 12.58 ± 3.19 | 72 |
| **Ticks** | 1.68 ± 0.54 | 36.36 | 0.96 ± 0.43 | 26 |
| *Dermacentor rhinocerinus* | 0.01 ± 0.01 | 1.37 | 0.04 ± 0.03 | 4 |
| *Haemaphysalis elliptica* group | 0.14 ± 0.07 | 6.85 | 0.18 ± 0.08 | 12 |
| *Hae. spinulosa* group | 0.05 ± 0.04 | 2.74 | 0 | 0 |
| *Hyalomma truncatum* | 0.77 ± 0.26 | 20.55 | 0.34 ± 0.13 | 1.43 |
| *Rhipicephalus follis/simus* group | 0.26 ± 0.22 | 5.48 | 0.40 ± 0.40 | 2 |
| **Mites** | 39.17 ± 5.30 | 87.01 | 17.42 ± 4.78 | 68 |
| *Androlaelaps marshalli* | 4.48 ± 0.71 | 67.12 | 2.56 ± 0.73 | 46 |
| *A. oliffi* | 30.44 ± 5.12 | 82.19 | 11.98 ± 4.26 | 56 |
| *A. taterae* | 0.12 ± 0.06 | 8.22 | 0.08 ± 0.08 | 2 |
| *A. theseus* | 1.99 ± 0.46 | 38.36 | 1.08 ± 0.39 | 26 |
| *Laelaps liberiensis* | 0.22 ± 0.14 | 6.85 | 0.02 ± 0.02 | 2 |
| *L. muricola* | 0.01 ± 0.01 | 1.37 | 0 | 0 |
| *L. simillimus* | 0.21 ± 0.21 | 1.37 | 0.08 ± 0.08 | 2 |
| *Ornithonyssus bacoti* | 0.01 ± 0.01 | 1.37 | 0 | 0 |
| *Pachylaelaps* sp.^P^ | 0.11 ± 0.05 | 6.85 | 0.08 ± 0.04 | 8 |
| Uropodidae sp.^P^ | 0.34 ± 0.16 | 12.99 | 0.02 ± 0.02 | 2 |
| Acaroidae sp.^P^ | 0.79 ± 0.78 | 2.60 | 0 | 0 |
| *Listrophoroides (A.) mastomys* | 0.74 ± 0.42 | 5.48 | 1.0 ± 0.98 | 4 |
| *Cheyletus zumpti* ^P^ | 1.73 ± 0.47 | 28.77 | 0.46 ± 0.22 | 14 |
| *Austromyobia* *forcipifer* | 0.03 ± 0.02 | 2.70 | 0.06 ± 0.04 | 4 |
| **Chiggers** | - | 33.77 | - | 38 |
| *Gahrliepia nana* | - | 6.85 | - | 8 |
| *Ascoschoengastia ueckermanni* | - | 0 | - | 2 |
| *Microtrombicula mastomyia* | - | 2.74 | - | 0 |
| *Microtrombicula* sp. | - | 1.30 | - | 0 |
| *Schoutedenichia dutoiti* | - | 1.37 | - | 6 |
| *S. horaki* | - | 2.74 | - | 4 |
| *S. lumsdeni* | - | 9.59 | - | 12 |
| *S. morosi* | - | 8.22 | - | 4 |
| *Trombicula walkerae*  *Taxon count includes all male and female individuals, ^#^Count for flea species based on male individuals only, ^P^Predatory feeding strategy. | - | 1.37 | - | 2 |

**Table S2.** Epifaunistic arthropod taxa and their infestation parameters recorded from *Gerbilliscus leucogaster* (*n* = 127) in natural and agricultural habitat types in Mpumalanga Province, South Africa, 2014–2020.

| Ectoparasite taxa | Natural | | Agriculture | |
| --- | --- | --- | --- | --- |
|  | Mean Abundance (±SE) | Prevalence (%) | Mean Abundance (±SE) | Prevalence (%) |
| **Fleas^*^** | **3.49 ± 0.44** | **75** | **3.12 ± 0.58** | **84.48** |
| *Xenopsylla bechuanae^#^* | 0.01 ± 0.01 | 1.47 | 0.02 ± 0.02 | 1.72 |
| *X. brasiliensis^#^* | 0.06 ± 0.05 | 2.94 | 0.05 ± 0.04 | 3.45 |
| *X. frayi^#^* | 3.41 ± 0.45 | 70.59 | 3.05 ± 0.58 | 81.03 |
| **Lice** | **15.78 ± 2.97** | **75** | **12.12 ± 2.35** | **77.59** |
| *Hoplopleura biseriata* | 0.01 ± 0.01 | 1.47 | 0.05 ± 0.03 | 5.17 |
| *Polyplax biseriata* | 15.76 ± 2.97 | 75 | 12.07 ± 2.36 | 74.14 |
| **Ticks** | **2.31 ± 0.66** | **48.53** | **0.34 ± 0.16** | **13.79** |
| *Dermacentor rhinocerinus* | 0.04 ± 0.03 | 4.41 | 0 | 0 |
| *Haemaphysalis elliptica* group | 0.24 ± 0.09 | 13.24 | 0.07 ± 0.04 | 5.17 |
| *Hae. spinulosa* group | 0.06 ± 0.05 | 2.94 | 0 | 0 |
| *Hyalomma truncatum* | 1.14 ± 0.54 | 35.29 | 0.26 ± 0.15 | 6.90 |
| *Rhipicephalus follis/simus* group | 0.56 ± 0.37 | 5.88 | 0.02 ± 0.02 | 1.72 |
| **Mites** | 37.71 ± 6.47 | 69.12 | 23.10 ± 3.32 | 93.10 |
| *Androlaelaps marshalli* | 3.40 ± 0.81 | 42.65 | 3.91 ± 0.57 | 75.86 |
| *A. oliffi* | 30.03 ± 6.06 | 60.29 | 13.64 ± 2.34 | 82.76 |
| *A. taterae* | 0.07 ± 0.06 | 2.94 | 0.14 ± 0.08 | 8.62 |
| *A. theseus* | 1.47 ± 0.43 | 26.47 | 1.71 ± 0.45 | 39.66 |
| *Laelaps liberiensis* | 0.24 ± 0.15 | 7.35 | 0.02 ± 0.02 | 1.72 |
| *L. muricola* | 0.01 ± 0.01 | 1.47 | 0 | 0 |
| *L. simillimus* | 0.28 ± 0.23 | 2.94 | 0 | 0 |
| *Ornithonyssus bacoti* | 0 | 0 | 0.02 ± 0.02 | 1.72 |
| *Pachylaelaps* sp.^P^ | 0.04 ± 0.03 | 4.41 | 0.16 ± 0.07 | 10.34 |
| Uropodidae sp.^P^ | 0.24 ± 0.17 | 7.35 | 0.19 ± 0.08 | 10.34 |
| Acaroidae sp.^P^ | 0 | 0 | 1.05 ± 1.03 | 3.45 |
| *Listrophoroides (A.) mastomys* | 0.34 ± 0.31 | 4.41 | 1.09 ± 0.87 | 3.45 |
| *Cheyletus* *zumpti* ^P^ | 1.25 ± 0.39 | 23.53 | 1.10 ± 0.44 | 20.69 |
| *Austromyobia* *forcipifer* | 0.01 ± 0.01 | 1.47 | 0.09 ± 0.05 | 5.17 |
| **Chiggers** | - | **22.41** | - | **47.05** |
| *Gahrliepia nana* | - | 3.45 | - | 11.76 |
| *Ascoschoengastia ueckermanni* | - | 0 | - | 1.47 |
| *Microtrombicula mastomyia* | - | 3.45 | - | 0 |
| *Microtrombicula* sp. | - | 0 | - | 1.47 |
| *Schoutedenichia dutoiti* | - | 1.72 | - | 4.41 |
| *S. horaki* | - | 5.17 | - | 1.47 |
| *S. lumsdeni* | - | 1.72 | - | 17.65 |
| *S. morosi* | - | 3.45 | - | 8.82 |
| *Trombicula walkerae* | - | 3.45 | - | 0 |

*Taxon count includes all male and female individuals, ^#^Count for flea species based on male individuals only, ^P^Predatory feeding strategy.
